# Cholecystocolonic Fistula Presenting With Gallstone Ileus: A Report of a Rare Surgical Case

**DOI:** 10.7759/cureus.93624

**Published:** 2025-10-01

**Authors:** Rishi Kela, Sanket Katara, Lakir Patel, Foram Modh, Vipul D Yagnik

**Affiliations:** 1 Surgery, Smt. Shardaben Chimanlal Lalbhai General Hospital, Ahmedabad, IND; 2 Surgery, Banas Medical College and Research Institute, Palanpur, Palanpur, IND

**Keywords:** bowel obstruction, ccf, cholecystocolonic fistula, enterolithotomy, gallstone ileus

## Abstract

Cholecystocolonic fistula (CCF) is a rare entity resulting from chronic gallstone disease. Here we describe a 60-year-old female who presented with abdominal distension, colicky pain, and constipation for a period of five days. Imaging studies revealed pneumobilia and small bowel obstruction. Exploration via laparotomy revealed a 3.5 cm gallstone lodged in the terminal end of the ileum and a fistulous tract from the gallbladder to the hepatic flexure of the colonic bowel. Surgical intervention consisted of enterolithotomy, cholecystectomy, and colonic resection with primary anastomosis. Fortunately, postoperative recovery was smooth and uneventful. Preoperative diagnostic challenges, the salience of imaging, and surgical intervention are emphasized in this case. As a brief background review, we searched the literature to outline the spectrum of the disease and the advancing methods to overcome CCF.

## Introduction

Cholecystocolonic fistula (CCF) is an uncommon complication of chronic cholecystitis, identified in 0.06-0.14% of patients undergoing cholecystectomy and in 0.1-0.5% of autopsy specimens [1]. It represents the second most common form of cholecystoenteric fistula after the cholecystoduodenal type, yet preoperative diagnosis remains rare, achieved in only 7.9% of cases [2]. The fistula typically develops through recurrent inflammation and adhesion of the gallbladder to adjacent bowel, with subsequent erosion into the colon [3].

The clinical relevance of CCF lies in its wide spectrum of presentations. While some patients remain asymptomatic, others experience nonspecific gastrointestinal symptoms such as chronic diarrhea and malabsorption, often related to the loss of bile salts [4,5]. More severe complications, including recurrent cholangitis or acute intestinal obstruction from gallstone ileus, may also occur [4,6]. Although a classical triad of pneumobilia, diarrhea, and vitamin K deficiency has been described, it is rarely observed in practice [7].

From a diagnostic perspective, CCF is clinically significant because its subtle and variable presentation frequently delays recognition. Computed tomography (CT) is the most useful imaging modality, with reported sensitivity exceeding 90% in some series, yet many cases are still diagnosed intraoperatively [2,8,9].

This report adds to the literature by describing a rare presentation of CCF complicated by gallstone ileus, in which a large gallstone migrated through a CCF and became impacted in the terminal ileum. The case underscores the diagnostic value of CT in identifying pneumobilia and the fistulous tract, highlights the surgical principles of combined enterolithotomy, cholecystectomy, and colonic resection, and emphasizes the importance of considering this uncommon pathway in patients presenting with small bowel obstruction. The aim of presenting this case is to raise awareness of this rare mechanism and provide guidance for timely surgical management.

## Case presentation

A 60-year-old woman with a history of gallstone disease presented with abdominal distension, colicky pain, and constipation for five days. Examination revealed a distended abdomen, diffuse tenderness, and sluggish bowel sounds without peritoneal signs. The patient’s routine pre-operative investigations, liver function tests, and CRP are summarized in Table 1.

**Table 1:** Laboratory findings in the patient with a cholecystocolonic fistula. Normal ranges are standard laboratory values; patient values are from the case described. AST: aspartate aminotransferase; SGOT: serum glutamic-oxaloacetic transaminase; ALT: alanine aminotransferase; SGPT: serum glutamic-pyruvic transaminase

Investigation	Patient Value	Normal Range	Interpretation
Hemoglobin	12.8 g/dL	12-16 g/dL	Normal
Total leukocyte count	11,200/mm^3^	4,000-10,000/mm^3^	Mild leukocytosis
Platelet count	2.3 × 10^5^/µL	1.5-4.0 × 10^5^/µL	Normal
Total bilirubin	0.9 mg/dL	0.2-1.2 mg/dL	Normal
Direct bilirubin	0.3 mg/dL	<0.3 mg/dL	Normal
AST (SGOT)	32 U/L	<40 U/L	Normal
ALT (SGPT)	28 U/L	<40 U/L	Normal
Alkaline phosphatase (ALP)	155 U/L	44-147 U/L	Mildly elevated
Serum albumin	4.1 g/dL	3.5-5.0 g/dL	Normal
C-reactive protein (CRP)	12 mg/L	<6 mg/L	Elevated
Serum creatinine	0.9 mg/dL	0.6-1.2 mg/dL	Normal
Blood urea nitrogen (BUN)	14 mg/dL	7-20 mg/dL	Normal

**Figure 1:**
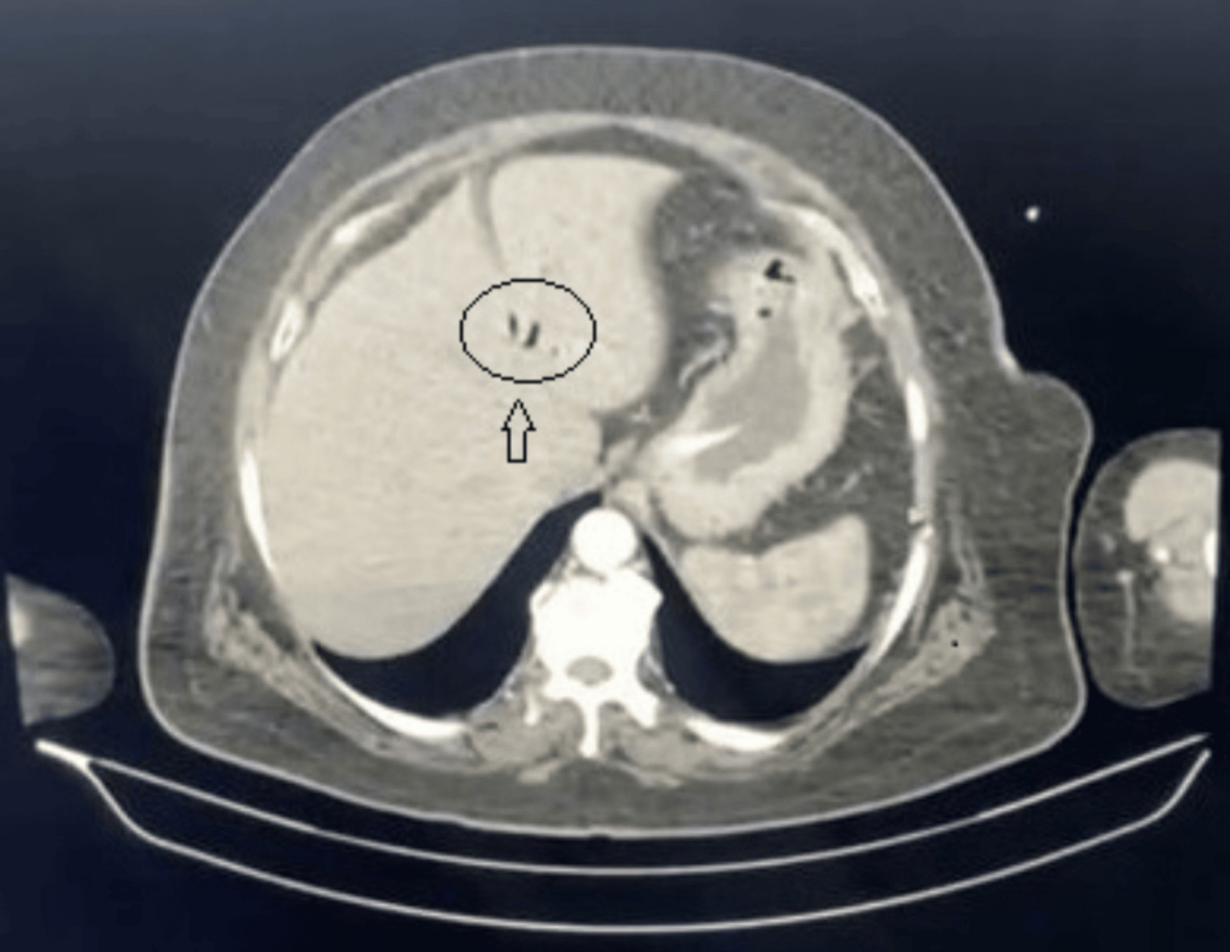
Axial computed tomography (CT) scan of the abdomen showing air within the biliary tree (pneumobilia, arrow).

**Figure 2:**
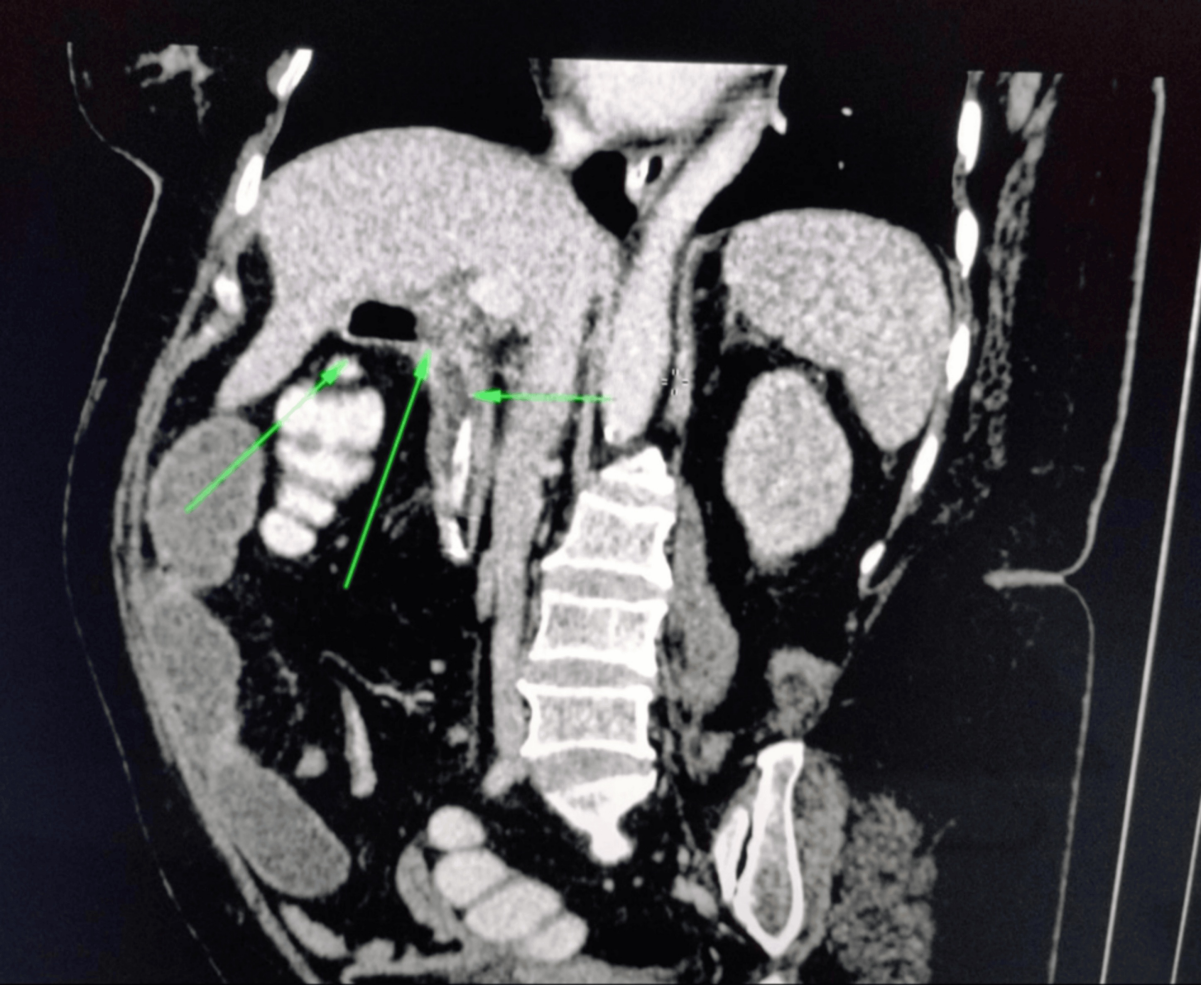
Coronal computed tomography (CT) scan demonstrating a fistulous tract between the gallbladder and colon (arrows), consistent with a cholecystocolonic fistula.

**Figure 3:**
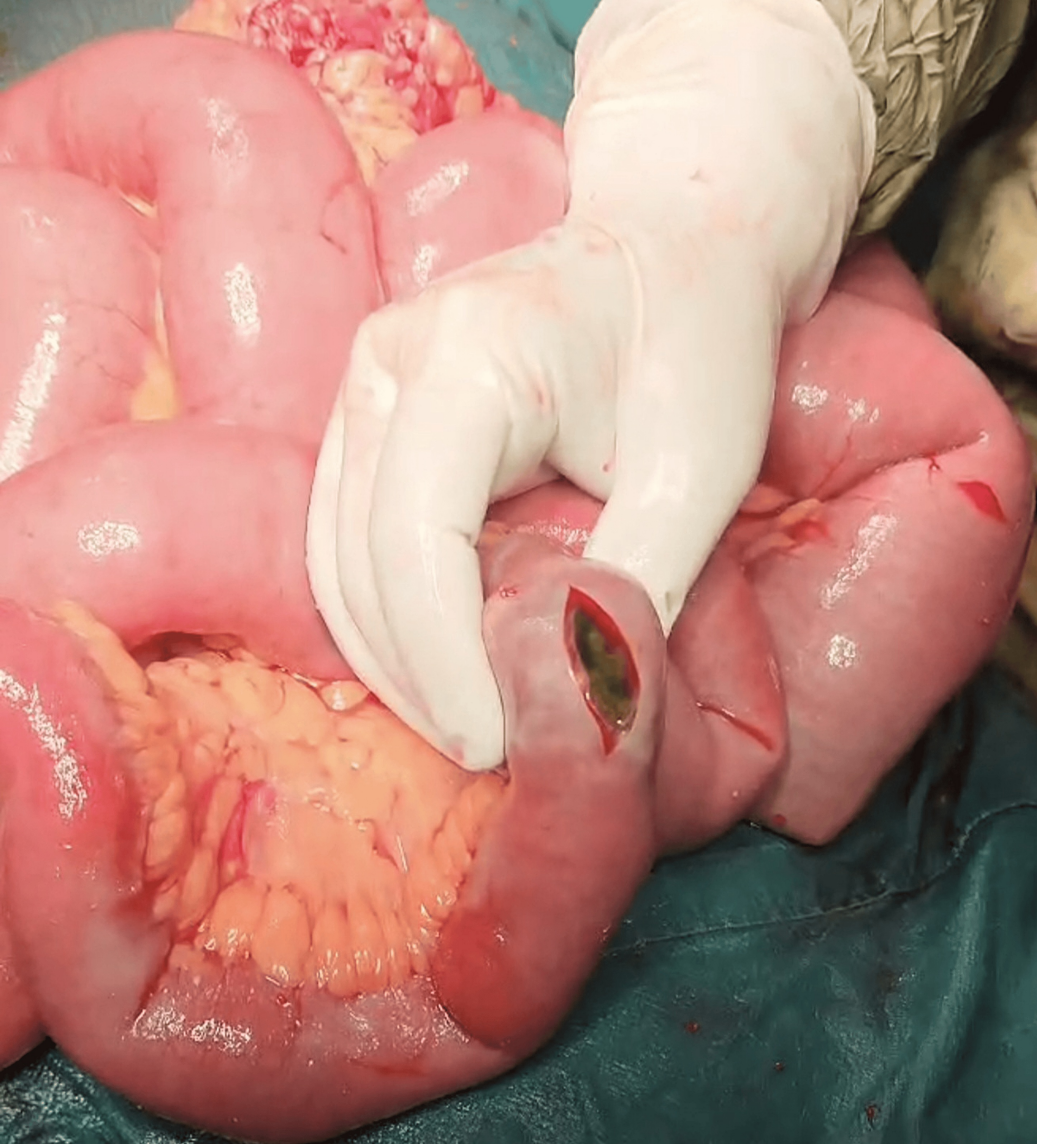
Operative photograph showing an enterotomy in a distended small bowel, with a large gallstone (dark brown-black) being extracted from the lumen.

**Figure 4:**
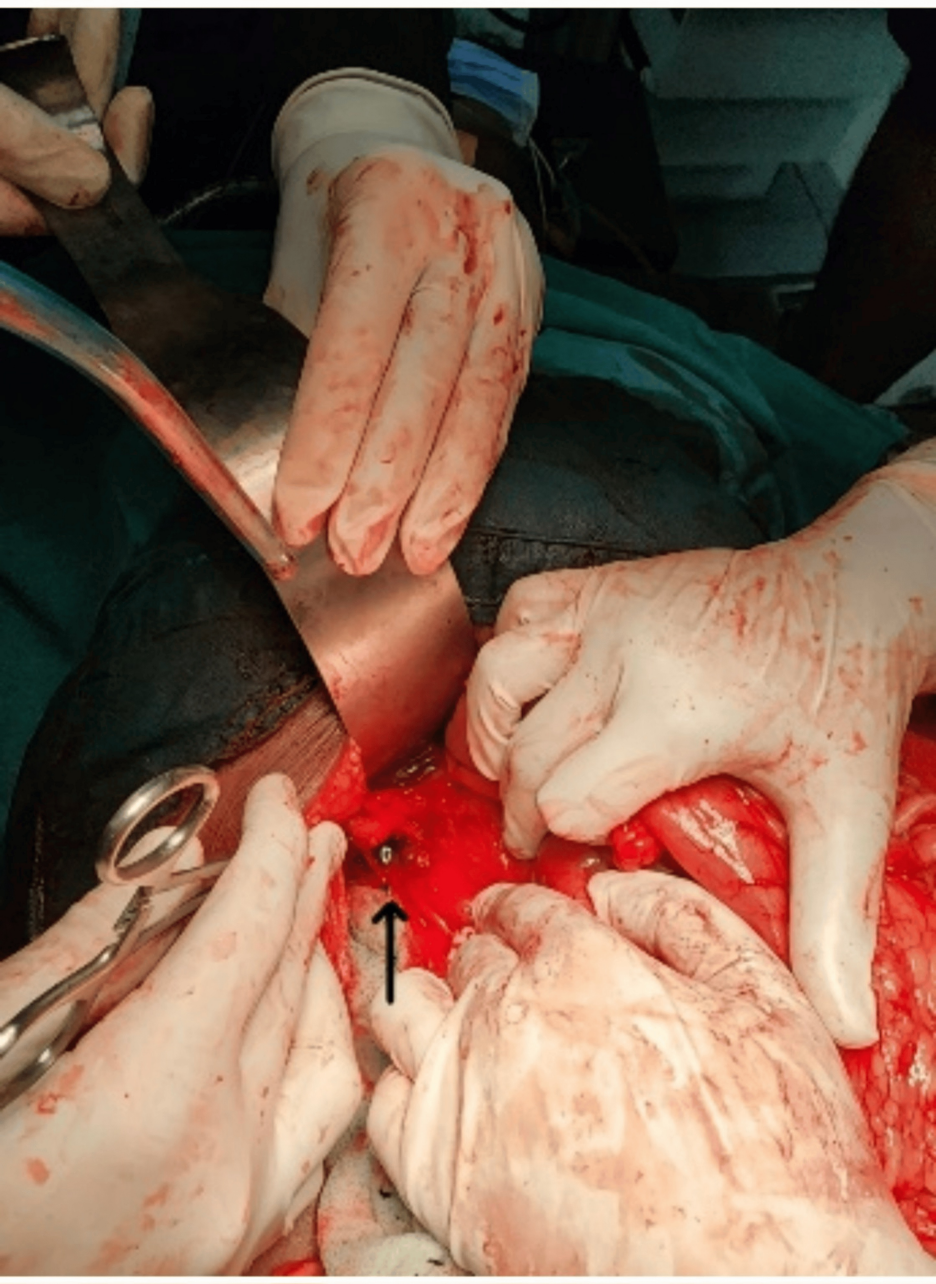
Intraoperative photograph showing a cholecystocolonic fistula (arrow).

Contrast-enhanced CT demonstrated dilated small bowel loops with pneumobilia (Figure 1) but failed to visualize the gallstone. The coronal image showed CCF (Figure 2). With suspected gallstone ileus, the patient underwent exploratory laparotomy. Intraoperative findings included a 3.5 cm gallstone impacted in the terminal ileum (Figure 3) and a CCF at the hepatic flexure (Figure 4). The small bowel was viable and intact.

She underwent enterolithotomy to remove the obstructing stone, cholecystectomy, and segmental colonic resection, including the fistula, followed by primary end-to-end colonic anastomosis. Postoperative recovery was uneventful, and she was discharged on day 7 in stable condition. The patient was in follow-up for six months without any complications.

## Discussion

Gallstone ileus typically develops following chronic cholecystitis, where ongoing inflammation causes the gallbladder to adhere to adjacent bowel, eventually resulting in fistula formation that allows gallstones to enter the gastrointestinal lumen and cause mechanical obstruction [10].

Although cholecystoduodenal fistula is the most common biliary-enteric fistula, colonic fistulas are uncommon, reported in 0-10.9% of series on gallstone-ileus-related fistulas [11]. In dedicated CCF literature, presentations range from clinically silent to nonspecific symptoms such as chronic diarrhea (often from bile-salt loss) and recurrent biliary infections [4,7]. It occurs most often in elderly women with longstanding gallstones. Our patient presented with intestinal obstruction, a less frequent manifestation compared to diarrhea or infection. Although CCF typically results in distal colonic passage of stones, a theoretical mechanism for small-bowel impaction would be retrograde migration across an incompetent ileocecal valve. Ileocecal valve incompetence can permit colo-ileal reflux, and the terminal ileum/valve is a recognized site of gallstone impaction; however, we found no published case directly confirming this pathway, and we therefore present it as a plausible hypothesis rather than a proven mechanism.

Laboratory abnormalities in our patient, including mild leukocytosis, elevated CRP, and mildly increased alkaline phosphatase (ALP), were consistent with an inflammatory biliary process. While nonspecific, these findings supported the clinical suspicion of gallstone-related pathology and, in conjunction with CT findings of pneumobilia and fistula, guided the decision for prompt surgical intervention.

CT has become the most valuable modality, capable of detecting biliary-enteric fistulas, pneumobilia, and gallstones with a reported sensitivity exceeding 90% in some series [2]. CT not only detects gallstones and fistulae but can also reveal associated conditions such as diverticular strictures. In contrast, CT is beneficial for assessing bowel perfusion, enabling recognition of ischemia or pressure necrosis from impacted stones and guiding timely surgical intervention. Magnetic resonance cholangiopancreatography (MRCP) can provide further anatomical detail when CT is inconclusive [4]. Endoscopic methods, including colonoscopy and endoscopic retrograde cholangiopancreatography (ERCP), may allow for direct visualization; ERCP, in particular, has been regarded as the most accurate preoperative test, although its feasibility is limited in acutely ill patients [12]. Barium enema may also demonstrate reflux of contrast into the biliary system [7]. Despite these modalities, many CCF cases are still diagnosed intraoperatively.

The surgical management of gallstone ileus remains a topic of controversy. Enterolithotomy alone is the simplest and safest option, particularly in elderly or unstable patients, but it leaves the fistula untreated and carries a risk of recurrence. One-stage surgery (enterolithotomy with cholecystectomy and fistula closure) provides definitive treatment but is technically demanding and associated with higher mortality [13-15]. Two-stage surgery (initial enterolithotomy followed by delayed biliary repair) offers a compromise, allowing stabilization before definitive surgery [14,15]. Most authors recommend tailoring the approach to the patient's comorbidities and intraoperative findings. Surgical management options with mortality are summarized in Table 2 [1,16].

**Table 2:** Comparison of surgical strategies for gallstone ileus and cholecystocolonic fistula. CCY: cholecystectomy

Surgical Strategy	Advantages	Disadvantages	Best Candidate	Reported Mortality
Enterolithotomy alone	Simplest, shortest operative time; useful in elderly/frail patients; lowest immediate operative risk	Does not treat fistula or gallbladder; risk of recurrent gallstone ileus (~5%); risk of recurrent biliary symptoms (~10%)	Elderly, frail, unstable patients with high comorbidity	≈11.7% [1,16]
One-stage surgery (enterolithotomy + cholecystectomy + fistula repair)	Definitive treatment; prevents recurrence; addresses gallbladder and fistula	Technically demanding; longer operative time; higher morbidity in frail patients	Fit, stable patients with good reserve and surgical support	≈16.9% [1,16]
Two-stage surgery (Step 1: enterolithotomy; Step 2: delayed CCY + fistula repair)	Balances safety and completeness; allows stabilization before definitive repair	Requires second surgery; risk of interval complications; requires compliance and follow-up	Younger or stable patients at risk of long-term biliary complications	≈10-12% (variable, lower if stabilized) [1]

Recent literature highlights the feasibility of laparoscopic management in selected cases, although conversion rates remain significant due to adhesions and inflammation [13,17-19]. Endoscopic interventions, such as holmium laser lithotripsy with stone basketing, have been described in high-risk patients unable to undergo major surgery [20]. In our patient, a one-stage open procedure was chosen because she was stable, providing definitive treatment consistent with current standards. Although laparoscopic management of CCF has been reported [13,16-18], open surgery remains the widely practiced approach in complex or uncertain intraoperative settings.

**Figure 5:**
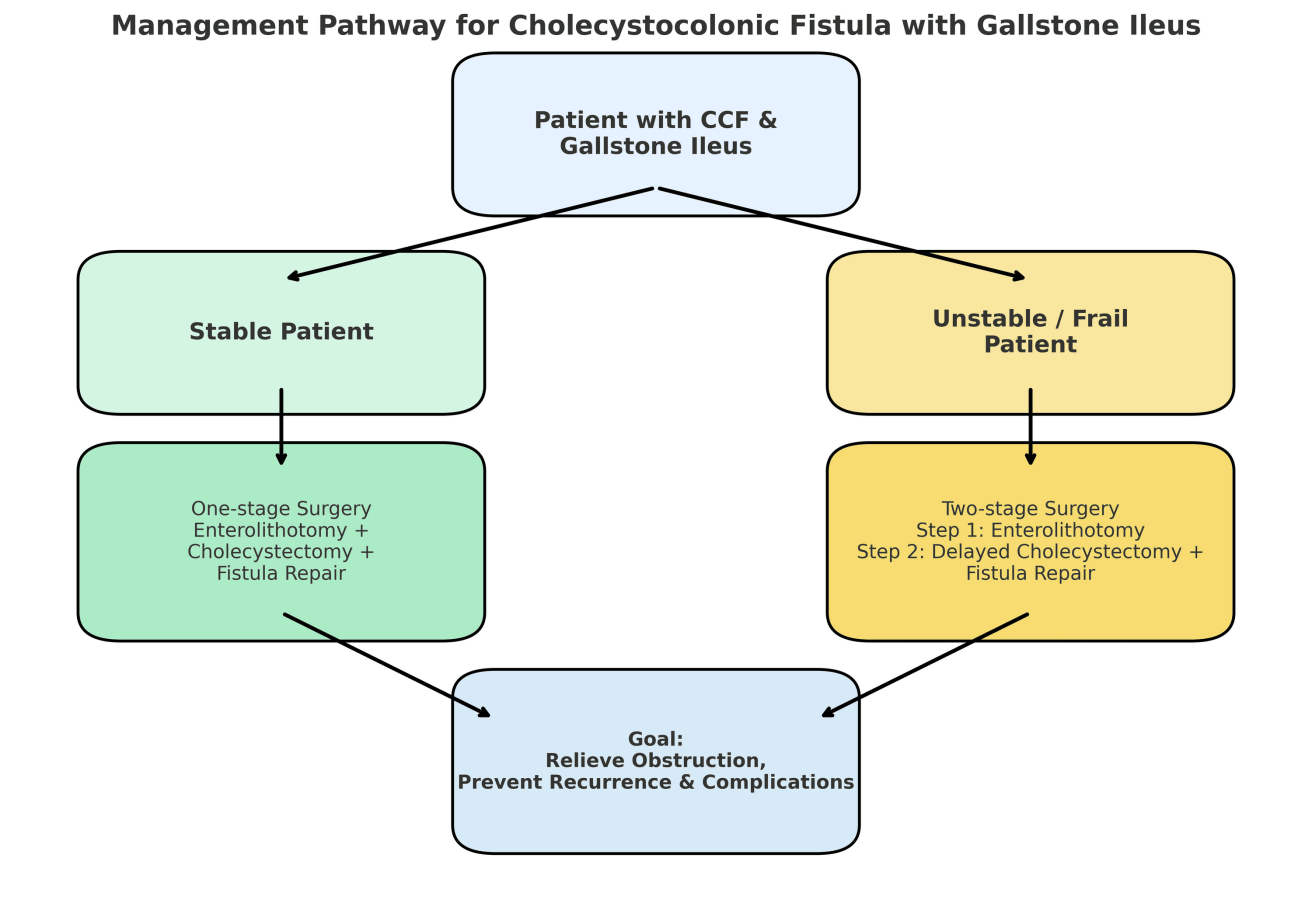
Management pathway for cholecystocolonic fistula (CCF) with gallstone ileus. Image credit: The authors.

Our patient recovered uneventfully and remained well at six-month follow-up, a favorable outcome compared with similar reports where recurrence or infectious complications have occurred. This case, therefore, reinforces the importance of early recognition, comprehensive surgical management, and structured follow-up in optimizing results for patients with this rare presentation. The management pathway for CCF is described in Figure 5.

## Conclusions

CCF is an uncommon complication of chronic cholecystitis and rarely presents with gallstone ileus due to ileal impaction. Our case highlights this atypical pathway, where a large gallstone migrated through a CCF and lodged in the terminal ileum, producing classical features of small bowel obstruction. Laboratory evidence of inflammation, combined with CT findings of pneumobilia and fistulous communication, guided timely surgical intervention. Although CT and ERCP can aid in preoperative diagnosis, many cases are still identified intraoperatively. A one-stage open procedure consisting of enterolithotomy, cholecystectomy, and segmental colonic resection with anastomosis provided definitive management, and the patient recovered uneventfully with a favorable six-month follow-up. This report highlights the importance of considering CCF in the differential diagnosis of elderly patients presenting with intestinal obstruction and emphasizes that one-stage surgery can be a safe and effective option in stable patients, achieving excellent outcomes.
